# Point-of-care community drug checking technologies: an insider look at the scientific principles and practical considerations

**DOI:** 10.1186/s12954-023-00764-3

**Published:** 2023-03-25

**Authors:** Lea Gozdzialski, Bruce Wallace, Dennis Hore

**Affiliations:** 1 Department of Chemistry, University of Victoria, Victoria, V8W 3V6 Canada; 2 School of Social Work, University of Victoria, Victoria, V8W 2Y2 Canada; 3 Canadian Institute for Substance Use Research, University of Victoria, Victoria, V8W 2Y2 Canada; 4 Department of Computer Science, University of Victoria, Victoria, V8W 3P6 Canada

**Keywords:** Drug checking, Test strips, Infrared absorption, Raman scattering, Surface-enhanced Raman scattering, Gas chromatography–mass spectrometry, Knowledge mobilization

## Abstract

Drug checking is increasingly being explored outside of festivals and events to be an ongoing service within communities, frequently integrated within responses to illicit drug overdose. The choice of instrumentation is a common question, and the demands on these chemical analytical instruments can be challenging as illicit substances may be more complex and include highly potent ingredients at trace levels. The answer remains nuanced as the instruments themselves are not directly comparable nor are the local demands on the service, meaning implementation factors heavily influence the assessment and effectiveness of instruments. In this perspective, we provide a technical but accessible introduction to the background of a few common drug checking methods aimed at current and potential drug checking service providers. We discuss the following tools that have been used as part of the Vancouver Island Drug Checking Project in Victoria, Canada: immunoassay test strips, attenuated total reflection IR-absorption spectroscopy, Raman spectroscopy from powder samples, surface-enhanced Raman scattering in a solution of colloidal gold nanoparticles, and gas chromatography–mass spectrometry. Using four different drug mixtures received and tested at the service, we illustrate the strengths, limitations, and capabilities of such instruments, and expose the scientific theory to give further insight into their analytical results. Each case study provides a walk-through-style analysis for a practical comparison between data from several different instruments acquired on the same sample. Ideally, a single instrument would be able to achieve all of the objectives of drug checking. However, there is no clear instrument that ticks every box; low cost, portable, rapid, easy-to-use and provides highly sensitive identification and accurate quantification. Multi-instrument approaches to drug checking may be required to effectively respond to increasingly complex and highly potent substances demanding trace level detection and the potential for quantification.

## Introduction

Drug checking is increasingly recognized as an important harm reduction strategy in a variety of situations ranging from testing at music festivals to community-based efforts and sanctioned safe consumption sites [1, 2]. These efforts often began with reagent-based testing, mostly within the rave and festival community, and have recently expanded to drug checking with portable and laboratory-based instruments [3, 4]. Simple tests such as using Ehrlich’s reagent for LSD, or lateral flow immunoassay test strips for fentanyl offer low cost, highly sensitive detection methods that are easy to use and provide immediate results, but are limited to providing a binary yes/no answer without any additional information on the composition of the sample. Ideally, drug checking provides the identity of all active components and cutting agents in addition to quantitative information on the potency by measuring and reporting the concentration of the active ingredients. Harm reduction practices have long existed amongst people who use drugs; many people describe seeking both qualitative and quantitative information through strategies such as test shots, or asking others who may have already consumed the substance about their experience [5]. Many service users and service providers have expressed that highly accurate quantitative information is essential for their perception of the usefulness of drug checking [6, 7].

To date, many instruments have been proposed and explored within the context of drug checking [8, 9] including infrared absorption spectroscopy [10–13], Raman scattering [14, 15], surface-enhanced Raman scattering (SERS) [16–18], nuclear magnetic resonance (NMR) [19, 20], ion mobility [21], electrochemical detection methods [22], and mass spectrometry (MS) either on its own [21] or coupled with gas chromatography (GC–MS) [14, 23, 24], liquid chromatography (LC–MS) [10, 14, 20, 23] or paper spray (PSMS) [25, 26]. Practically, there is often a trade-off between accuracy, cost, portability, speed, and ease-of-use including whether specialized training is required. Furthermore, there is generally a compromise in these same categories between portable and lab-based instruments using the same underlying principles (e.g., portable GC–MS verses laboratory-based GC–MS) [27]. In some cases, hand-held instruments are desirable; in other cases this is not a strict requirement but instruments need to be small, lightweight, and robust enough that they can withstand frequent moving between locations. This includes transportation to festivals, and services rotating between several overdose prevention sites within a city [6, 28]. In addition to being larger and requiring a permanent stable location, laboratory-based instruments may also have site requirements including high voltage, the need for ventilation, the availability of compressed gases, and stringent environmental temperature and humidity control. With these considerations in mind, many drug checking projects use instruments such as portable IR spectrometers in combination with test strips for on-site testing [12, 29]. Such sites are often affiliated with a laboratory-based service for subsequent confirmatory testing [8, 12, 14].

There are significant challenges in understanding the pros, cons, and considerations for particular technologies within unique harm reduction sites, and communicating such information within a service. Further challenges arise in recognizing and predicting the ultimate potential of certain technologies given additional research and development. Recent reviews have provided a comprehensive overview of several analytical instruments and have discussed their applicability to harm reduction drug checking [8, 30–32]. The value and, in some cases, necessity of a multi-instrument approach to drug checking has also been recognized [31]. Yet, the right combination of instruments requires a comprehensive needs assessment that depends on many factors and stakeholders [3, 30, 33, 34]. As drug checking expands within harm reduction interventions, and as an active field of research, there is a growing need for a more in-depth explanation of the underlying technologies within this context. Furthermore, the traditional roles of operating instruments and interpreting data (i.e., technician role) and communicating results (i.e., harm reduction role) are rapidly becoming blended [29, 35]. Such knowledge provides the missing link between theory and practice that can best ensure service quality at drug checking sites, contribute to the body of practice-based evidence informing drug checking, and advance drug checking research through experiential learning [35].

This perspective is aimed at the modern drug checking service provider who has developed considerable expertise in their craft; performing measurements, interpreting data, and presenting results embedded in the principles of harm reduction [36, 37]. We have centred our presentation around common questions regarding the strengths, limitations, and possibilities of such instruments, and expose the scientific foundation that give further insight into the answers. The first section, “Background on some drug checking technologies”, introduces several point-of-care technologies and provides insight into some of the principles that influence their practical application in drug checking. For each analysis method, we provide a brief background on its history as a harm reduction drug checking technology, basic principles behind the technology, and a brief review of the science behind these technologies. The selection of scientific topics has been curated from questions that frequently arise while operating our own drug checking service [33]. Such questions also echo many themes that have evolved from the growing body of drug checking literature regarding the capabilities, limitations, and challenges of particular technologies. In the subsequent section “Illustrations from an active drug checking service”, we present four case studies of different drug mixtures received and tested at our service. Here we provide a step-by-step walk-through for a practical comparison of data from several different instruments acquired on the same samples.

## Background on some drug checking technologies

### Test strips

Test strips, also known as lateral flow immunoassays, provide a simple yes/no answer to whether a trace amount of a particular compound is present in a sample [38, 39]. The test strips commonly used for fentanyl and benzodiazepine screening in drug checking were originally designed for use with urine samples. Immunoassays offer a convenient, fast, and low-cost testing method, useful for outreach and at-home use [38, 40–42]. They also remain an important tool in many drug checking services [33, 43–45], even when analytical instruments are also employed. In the case of fentanyl test strips, their on-going use is also largely attributed to their reliability and low limit of detection (about 0.150 µg/mL) compared to many analytical instruments [17].

#### Basic principles

**Fig. 1:**
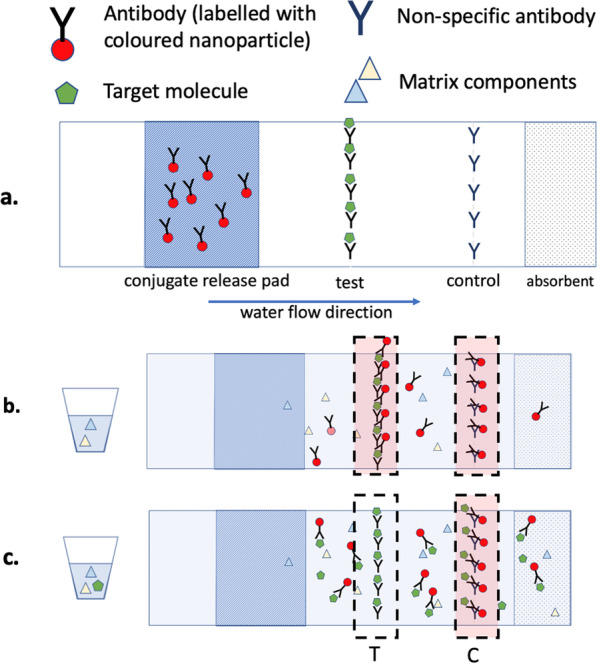
**a** Schematic of a competitive lateral flow immunnoassay dipstick, **b** Example of a fentanyl test strip where the both test and control line appears red, indicating a negative result, and **c** example of a fentanyl test strip where the control line appears red and test line is absent (inhibited), indicating a positive result due to fentanyl binding the labeled antibodies and preventing their interaction with the test line

Most commercially available lateral flow immunoassays for drug detection are based on the principles of competitive binding [46–48]. Here the absence of a test line indicates a positive result, in contrast to the sandwich assays where the appearance of a test line indicates a positive result (e.g., pregnancy tests, COVID-19 rapid tests). Immunoassays are all about the interaction between antibodies and antigens (i.e., the target molecule that binds to antibodies) [49]. In this example, fentanyl is the antigen. Figure 1a demonstrates key components of a strip test, including the pad that contains unbound gold-labeled antibodies specific to fentanyl, a bound row of the same antibodies and a control line with bound nonspecific antibody. In the scenario illustrated in Fig. 1b, no fentanyl is present in the solution (a negative result). Upon dipping the strip in the solution, the water will dissolve the antibodies from the pad. Since no fentanyl has bound, those antibodies are free to bind the test line resulting in the appearance of a red line. Since there are an excess of the antibodies flowing up with the water, they are also able to bind the control line and appear red. The presence of the control line indicates that the test strip has worked as expected, i.e., the water flowed properly and carried the antibodies. In the scenario illustrated in Fig. 1c, fentanyl is present above a certain threshold (a positive result). Here fentanyl, the antigen, will bind the unbound gold-labeled antibodies as the water flows up the test strip. Since these antibodies have been inhibited by fentanyl, they are not available to bind on the test line and therefore pass through. The control line, however, remains nonspecific; it will bind regardless of whether fentanyl is bound to the antibodies or not and should appear red.

#### Practical considerations

Immunoassay test strips have been widely adopted in many applications due to their simplicity and reliability [50, 51]. However, nuances still exist in the use of test strips within the context of drug checking [39, 52, 53]. Some common challenges that need to be accounted for when using test strips include: (1) false positives, where out-of-class compounds with structural similarities to the target class (e.g., opioid class) can result in high frequency of false positives; (2) false negatives, where the structural diversity of drugs within the same class (e.g., benzodiazepines), make it increasingly difficult to manufacture a single assay to confidently screen for all target substances without compromising selectivity; (3) subjective results: while manufacturers recommend to interpret the presence of a test line, regardless of its intensity, as a negative result it has been suggested that a more liberal interpretation is necessary, such as clear positive, positive, weak positive/ambiguous and negative; and (4) communication: knowing the correct way to communicate such results may be context-specific.

Many of the challenges listed above have been encountered at our drug checking service. In the case of fentanyl test strips, it is known that too much sample in solution causes false-positive results when that sample contains crystal meth or MDMA [54]. In the case of benzodiazepine test strips, etizolam, a benzodiazepine-related compound, has been shown to interact poorly [16, 55], resulting in a false-negative screen for benzodiazepines.

**Table:** 

What does it mean when the line appears faded?	
If the concentration of the target compound approaches the limit of detection, the intensity of the test line is generally correlated with concentration [54] and therefore may appear faded.

**Table:** 

Does this mean that test strips results can provide information on the concentration of the drug?	
Technically yes, however, this is not readily interpreted in terms of a concentration that translates into the drug potency. That connection requires more sensitive detection of the line color than can be gauged by eye, and a precise control of the sample mass and solution volume. Such careful preparation and digital read out is typically not the intent of test strip drug checking. Furthermore, solution pH, target drug solubility, and the presence of other drugs or cutting agents might affect binding, reaction times, and therefore the intensity of the test line [54, 56]. As a result, people utilize test strips for the qualitative information they readily provide.	

### ATR–IR spectroscopy

Infrared (IR) absorption spectroscopy is rapidly becoming one of the most widely used instrumental methods for drug checking on account of its relatively low cost, ease of operation, speed, minimal sample preparation requirements, and the availability of libraries (open source and commercial) containing thousands of drug components including cutting agents [8, 57–61]. IR spectroscopy can identify a wide range of compounds, but it has limited sensitivity in comparison with immunoassay test strips. Recent assessments of the suitability of IR spectroscopy for community drug checking focus on the detection and quantification of fentanyl and other compounds found in the opioid drug supply [10, 11, 62]. People with a limited background in science have been successfully trained to operate IR spectrometers; however, concerns remain around the possibility of misinterpreting data, as well as failing to recognize, and communicate, the limitations of such instruments [10, 13, 29].

#### Basic principles

**Fig. 2:**
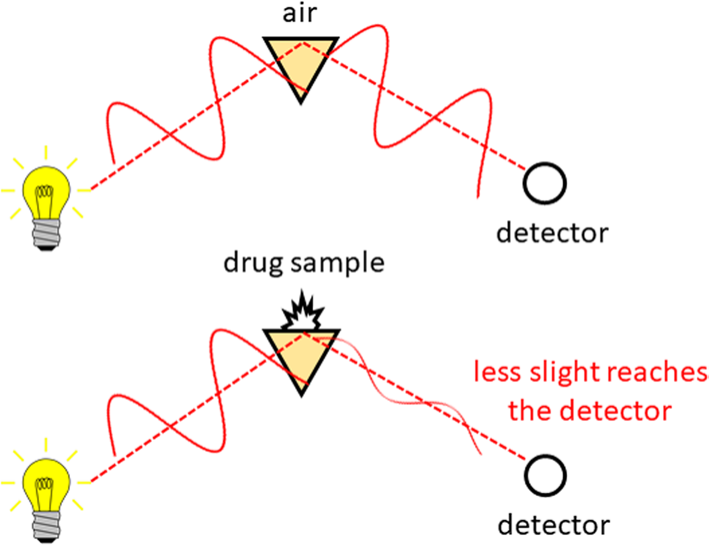
In an attenuated total internal reflection (ATR) absorption measurement, the sample is pressed against a prism, and infrared light is reflected from the surface. The ratio between measurements performed with and without the drug sample results in a spectrum that can be used to identify and quantify the components in a mixture

IR absorption refers to a family of techniques with the same basic operating principle. An IR light source is depicted as the lamp in Fig. 2 and typically emits in the frequency range of 500–5000 cm$^{-1}$, or the equivalent wavelength range of 20,000–2000 nm. The ratio of the light intensity before and after sample interaction is related to how much IR light is absorbed by a drug sample. Ultimately, an IR spectrum is a plot of the degree to which the light has been absorbed (the absorbance) as a function of the IR frequency.

Over the past several decades, nearly all IR absorption techniques make use of an instrument that is based on splitting and recombining the IR beam in an optical configuration known as an interferometer [63]. Subsequent Fourier transformation (FT) of the raw data provides the frequency spectrum. This is the basis for the acronym FTIR, referring to Fourier transform IR spectroscopy. The terms IR, IR absorption, and FTIR are therefore equivalent in this context. There are several possible sampling configurations for an IR experiment; most spectrometers used in drug checking reflect IR light through a small IR-transparent prism against which the sample is pressed [59, 60]. This method is known as ATR–IR, with the acronym describing the physical phenomena of attenuated total internal reflection (ATR) [64].

**Fig. 3:**
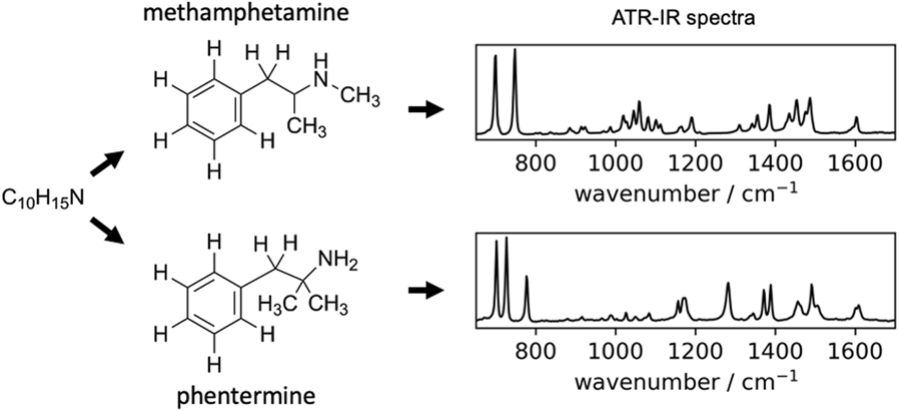
The structure of two compounds methamphetamine and phentermine, illustrating the different connectivity of the carbon framework, and location of the hydrogen and nitrogen atoms despite the same molecular formula. The similar, yet unique IR fingerprint is shown for each compound

All subsequent analysis to identify the components in the drug mixture is based on characteristic molecular vibrations in this frequency spectrum, originating from the structure of chemical bonds in the constituent molecules [65]. As an example, the chemical structure of methamphetamine is shown in Fig. 3. Other molecules with the same number of atoms, but different connectivity (for example, phentermine, Fig. 3), will have the same number of vibrations (termed modes), but they will have different characteristic frequencies and intensities. As a result of this molecular specificity, the IR spectrum acts as a fingerprint that can be searched against a database to identify which substances are present.

#### Practical considerations

It is often accepted that IR absorption methods can determine most significant components (active ingredients as well as cutting agents) in a mixture, as long as each one is at least $\approx 5$% of the overall mixture [28, 43, 66]. Although this is a reasonable estimate, in many cases such a hard-and-fast rule breaks down.

**Table:** 

What are the challenges associated with detecting components of drug mixtures using IR spectroscopy?	
(1) Any components present in low amounts will not absorb a significant enough fraction of the IR light to be detected. (2) Some molecules are difficult to distinguish from other species that have similar IR fingerprints, especially in low concentrations. (3) Successful identification relies on a library entry for each compound present.	

Identifying compounds at concentrations near the limit of detection is subject to technician experience and confidence, ultimately resulting in subjective and variable results. The small IR beam penetration depth also poses additional sampling challenges and contributes to uncertainty for heterogeneous mixtures (i.e., samples that might not be thoroughly mixed) [67].

**Table:** 

How much sample is required for an ATR–IR measurement?	
Although the sampling depth is roughly 0.002 mm, in order to ensure that the approximately 2 mm $\times$ 2 mm are of the crystal is covered, one uses approximately 1 mg of sample.

**Table:** 

How much of the sample is actually probed?	
Regardless of how much sample is placed on the spectrometer, the resulting IR spectrum depends only on the very small amount of sample right at the surface of the ATR crystal. Using the numbers above provides a volume of 2 mm $\times$ 2 mm $\times$ 0.002 mm = 0.008 mm$^3$. Assuming a density of 1.23 g/cm$^3$ (based on caffeine, for example), this corresponds to a mass of approximately 10 µg, or 1/100$^{\textrm{th}}$th of a milligram.	

**Fig. 4:**
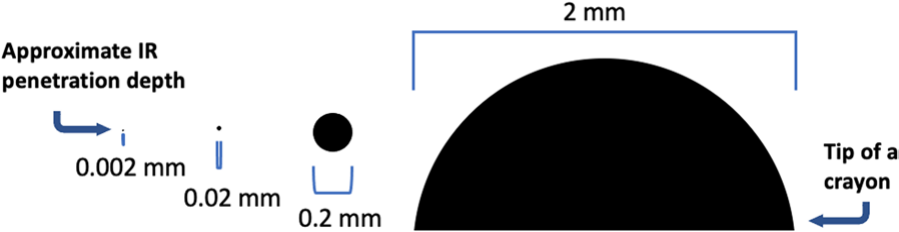
Graphical representation of the approximate sample penetration depth of 0.002 mm in ATR–IR absorption spectroscopy

**Fig. 5:**
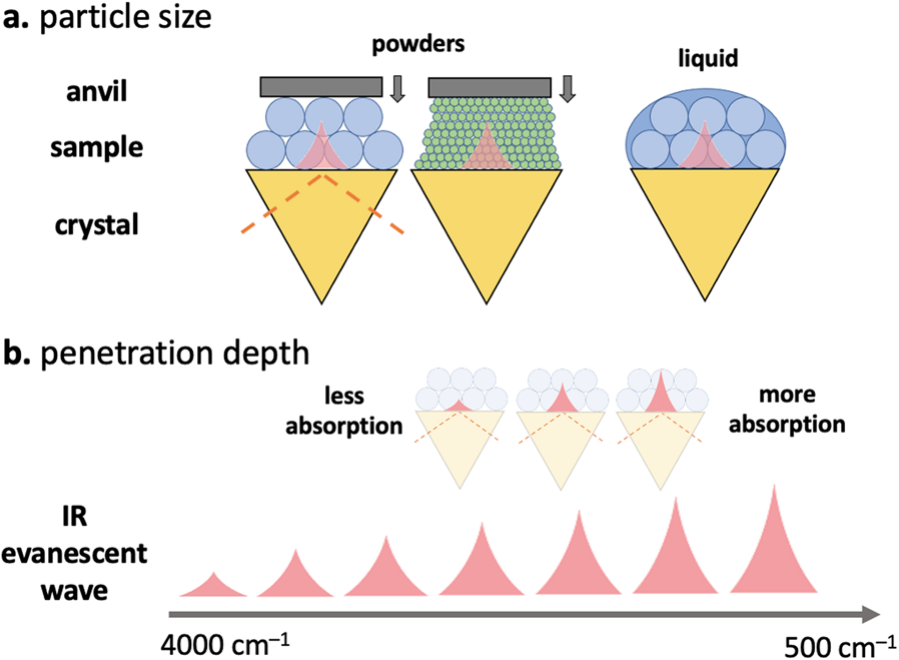
**a** A comparison of the sampling when powders and liquids are placed in contact with an ATR crystal. In the case of powered samples, there is a challenge in creating a close enough contact to ensure sufficient absorption of IR light since the particles may be loosely packed creating air gaps. Finer powders mitigate this issue as smaller particles can pack closer. **b** The wavelength dependence of the IR penetration depth further complicates the various packing scenarios, and ultimately affects the mixture analysis

Since the probing volume is so small (graphical representation shown in Fig. 4), obtaining quality ATR–IR spectra relies on close contact of the sample with the ATR crystal. This is why we have to apply pressure to solid samples using an anvil, but not to liquids, to collect the spectra. Figure 5 illustrates some scenarios at the crystal–sample interface that might affect the reproducibility of the resulting IR spectra. For example, there is a limit to how closely particles of different sizes and shapes can be packed, resulting in air gaps (Fig. 5A) [68]. The depth of the IR beam penetration into the sample also depends on the optical properties of the sample and crystal, IR wavelength (Fig. 5B), and angle of incidence (typically fixed at 45$^\circ$) [64, 67].

**Table:** 

Why do ATR–IR spectra often have a sloping baseline?	
Although most software performs a simple correction for the wavelength-dependence of the probing IR light, the optical properties of the sample are more difficult to account for, especially with mixtures, and so the penetration depth still varies somewhat with wavelength.

Furthermore, inhomogeneous powders and a distribution of particle sizes can affect the shape of the baseline and also contribute to peak shifting and broadening [69].

**Table:** 

What are challenges associated with determining concentration of a particular component in a drug mixture?	
For starters, it may be difficult to *identify* the component as a result of any one of the reasons highlighted above. In the event of a successful library match, quantification is still challenging as spectral unmixing relies on the assumption that IR spectra of each of the pure components will be ideally superimposed to create the spectrum of the mixture. In practice, this often does not occur as a result of interactions between molecules in a mixture (often termed matrix effects), uncertainty in the optical constants of the mixture, and the various baseline artifacts described above.

These same challenges apply when discussing the purity of a given drug sample. In many cases, only one component is identified using IR spectroscopy. However, in general the purity (e.g., how close the drug is to being 100% one ingredient) of a compound can never be quantitatively determined using techniques with limited sensitivity, such as IR spectroscopy. For example, a combination of precursors, by-products, solvents, and other impurities may be present in low concentrations. In addition, precursors or by-products from the drug synthesis may share many structural, and therefore spectral similarities, to the major drug product.

### Raman spectroscopy

Raman spectroscopy is another vibrational spectroscopy method that provides molecular identity information, but based on the way a molecule scatters light as opposed to the way a molecule absorbs light in IR spectroscopy [70]. The utility of Raman spectroscopy in drug checking is frequently compared to that of ATR–IR due to their complementary nature [71, 72]. They also share many favorable characteristics: both techniques are quick, nondestructive, can be implemented with portable and robust hardware, and can identify a wide range of drugs, cutting agents and mixtures. Use of Raman spectroscopy has been reported for festival drug checking [14, 73, 74], as well as in community drug checking [9, 15, 75]. However, similar to IR, it lacks the sensitivity required for trace detection.

#### Basic principles

**Fig. 6:**
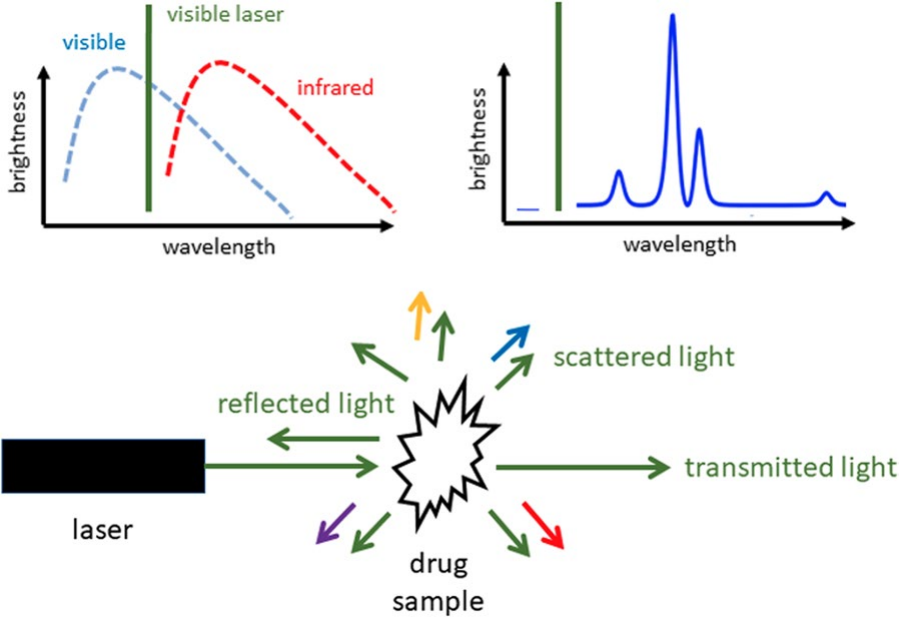
In a Raman scattering measurement, a powder or liquid sample of the drug mixture is illuminated with a laser beam. Some of the reflected light appears at wavelengths (colors) different from that of the laser, and these wavelengths are characteristic of the constituent molecules in the sample

In Raman spectroscopy, a laser that is typically in the visible region such as 488 nm (blue), 532 nm (green), or 633 nm (red) is focused onto a solid, powder, or liquid sample. Most of the light is either transmitted, absorbed, or reflected as shown in Fig. 6. However, a small quantity (typically less than 1%) is scattered in all directions [70, 76]. The experimental challenge is to collect this scattered light and analyze its spectrum. A small portion of this scattered light will be shifted in wavelength after interacting with the molecules in the sample; this is known as Raman scattering [77]. The spectrum of the Raman scattering provides a chemical fingerprint of the molecular vibrations of the sample components in much the same way as an IR absorption spectrum and can be directly analyzed [78] and compared against a reference database for identification. The relative intensity of vibrational modes will be different than those in the IR although the frequencies are the same [79]. For this reason, one needs a database that contains dedicated Raman libraries.

#### Practical considerations

The appearance and quality of Raman spectra depend on the instrument configuration, such as laser power, laser wavelength, light focusing, and spot size [76]. In general, most of the main challenges listed for IR spectroscopy above also apply (e.g., overlapping peaks, low sensitivity, requirement for libraries). Specific advantages of Raman include through-bag and through-barrier capability [80] and minimal interference with water [81].

**Table:** 

Why is Raman spectroscopy less popular than IR spectroscopy in the drug checking community?	
A major shortcoming of Raman spectroscopy is that, for certain samples, the weak Raman scattered light is overwhelmed by fluorescence that is typically orders of magnitude brighter than the Raman scattering.	

The fluorescence of a particular molecule (such as heroin) is predicable, but significant fluorescence may also originate from unknown trace impurities. Spectral processing can sometimes successfully subtract fluorescence background, allowing accurate qualitative and quantitative detection. However, if the fluorescence is too strong, no chemical information can be obtained.

**Table:** 

What can be done to mitigate the effects of fluorescence?	
The best approach is to select a laser wavelength that does not result in significant fluorescence. For white powders, fluorescence is generally avoided by choosing a longer wavelength laser. For example, moving from 532 nm to 785 nm to 830 nm. Typically, portable Raman spectrometers are only equipped with a single-wavelength laser. One can imagine that it is near-impossible to select one wavelength that minimizes fluorescence for every single molecule when measuring such a diverse range of substances.	

Colored samples (for example, opioid mixtures that have been dyed purple) are particularly challenging, as it is difficult to select a laser with a long enough excitation wavelength [76]. Near-infrared excitation at 1064 nm has been demonstrated to be a successful approach for such samples, with the compromise of lower sensitivity [82]. Other strategies to reduce fluorescence are to dilute or photo-bleach the sample [76].

**Table:** 

When might one choose Raman over IR? Is there a benefit to using both?	
In some cases, common mixtures that may be heavily overlapped in their IR spectrum might show unique and isolated peaks in the Raman spectrum, or vice versa; some of these cases will be discussed later. An obvious example is water. Water is a very weak Raman scatterer and so drugs dissolved in solution or that have absorbed moisture can more easily be measured without significant interference. In contrast the IR spectrum suffers from strong absorption by water [83].

**Table:** 

How does Raman spectroscopy work through a clear bag?	
Since Raman spectrometers typically use a visible light source, the measurement can be performed through transparent bags as they do not absorb visible light. Similar to water, some common and convenient materials such as polyethylene (a very common plastic) and glass are weak Raman scatterers relative to most drug molecules [76].	

Spatially offsetting the collection of Raman signal has found use in measuring tablets where coatings may interfere in measuring the active ingredient [76, 84], or allow to get a more bulk sampling of drug. Many handheld Raman spectrometers benefit from a large spot size (about 2 mm in diameter) and therefore, a larger sampling area; an advantage when considering heterogeneous drug mixtures.

Raman spectroscopy can also be used for quantification, meaning the Raman signal of a substance within a mixture can be related to its concentration.

**Table:** 

What is the limit of detection (LOD) for Raman spectroscopy?	
The limit of detection of specific compounds depends on details of the instrument hardware, the extent to which the fluorescence can mitigated, and how much the target molecule Raman signature differs from that of other components in the mixture, both in terms of relative intensity and the characteristic frequencies of the bands.	

Studies have shown, for example, that for cocaine the limit of detection can range from 10% when cut with inositol to 40% when cut with paracetamol [85]. In another study, the LOD for heroin in a mixture was found to be as low as $\approx 5$% [86].

### Surface-enhanced Raman spectroscopy

Surface-enhanced Raman scattering (SERS) has been developed in order to detect ultra-low concentrations of analytes [87–90]. This has enabled the detection of trace components in drug mixtures [91–94]. SERS may also alleviate the other primary challenge with Raman spectroscopy, fluorescence [76]. SERS has recently been explored for application in harm reduction and drug checking exclusively in the detection and quantification of trace compounds in mixtures, most commonly opioids [16, 94]. Although SERS is not widely employed in drug checking, it is gaining attention on account of its potential for trace detection.

#### Basic principles

The general principle behind SERS is that the Raman response may be amplified by a factor ranging from ten thousand to a million when the samples are placed near metal surfaces with sharp edges [95]. A particularly simple and effective approach is to use a solution of metal nanoparticles in solution. When the nanoparticles aggregate after the addition of a suitable salt (e.g., NaCl, MgSO$_4$), the distance between neighboring particles becomes small, and the Raman signal is greatly enhanced if drug component molecules can be trapped in that region [93, 96, 97]. The precise mechanism by which the Raman response is amplified continues to be of research interest in the chemistry and physics communities, but appears to be sensitive to the nature of the nanoparticles, the solution conditions, and the specific target molecules [76, 95, 98]. For this reason, libraries prepared for standard Raman scattering are generally unsuitable for searching using SERS data and must be tailored to the specific test conditions.

#### Practical considerations

SERS is poised to fill a gap in point-of-care drug checking by offering trace-level identification using relatively simple sample preparation and instrumentation. However, the signal acquired from SERS can be complex and difficult to interpret.

**Table:** 

Why is SERS challenging to implement as a widespread screening tool?	
There is a significant amount of method development and validation needed for each specific application; the behavior of a specific analyte, or mixture of drugs is hard to predict without comprehensive testing, research and development. The optimal choice of substrate, laser wavelength, aggregating agents and analyte concentration might differ for different target analytes.	

**Fig. 7:**
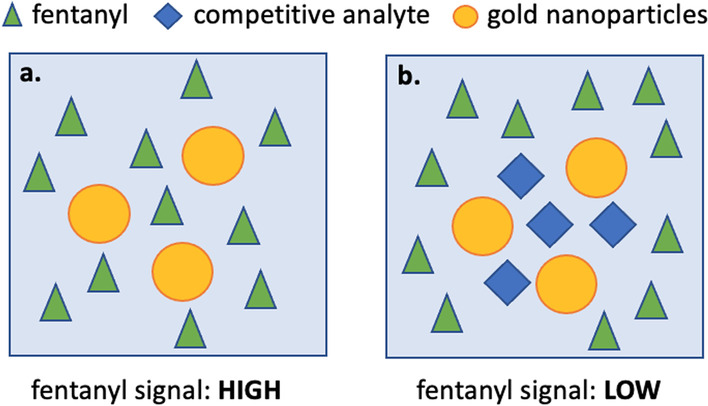
Illustration of the challenges with competitive adsorption in SERS. **a** and **b** represent two solutions with the same concentration of fentanyl, however **b** is in the presence of a competitive analyte with greater affinity to the gold nanoparticles than fentanyl. Despite having the same concentration of fentanyl in both solutions, the SERS spectrum may look significantly different. In the extreme case, fentanyl may give no signal in scenario (**b**)

For example, the SERS signal for substances such as fentanyl has been shown to decrease when the concentration is too high [93]. Furthermore, as mixtures become more complicated, the relationships between analyte concentration and signal intensity are less predictable. A case in point, as the relative concentration of fentanyl to etizolam increases, it becomes harder to detect etizolam [16]. Such behavior is often attributed to competitive adsorption for the gold nanoparticles [16, 98]. Figure 7 further illustrates this challenge, where the same concentration of fentanyl alone and in the presence of a competitive analyte (even a contaminant in trace amounts) might give dramatically different results.

On the other hand, if a substance is in very low concentration relative to a bulk cutting agent, but has a significantly higher affinity for the gold nanoparticles, it could still produce a strong signal without being drowned out by the major components. There is also interest in functionalizing nanoparticles to target specific molecules to achieve selective amplification in a mixture [99].

**Table:** 

Is SERS quantitative?	
SERS signal from an analyte typically follows some relationship with concentration, however it can be fairly nonlinear due to the challenges described above. Some strategies have been employed for more reliable quantification with SERS, such as using internal standards or standard addition methods [100]. Again, these methods require significant method development and calibration for particular molecules of interest.	

Another advantage of SERS in drug checking is that it potentially reduces the fluorescence, a major challenge in testing colored drug samples with traditional Raman spectroscopy [76].

**Table:** 

Why is fluorescence reduced in SERS experiments?	
SERS is not immune to fluorescence. However, the extremely low concentrations of analyte as well as quenching from the metal SERS substrate itself within the hot spots may result in reduced fluorescence [76, 95].	

Raman spectroscopy, in combination with SERS, is uniquely positioned to provide spectral data for the identification of high concentration bulking agents, as well as actives and trace components using a single instrument.

### Portable gas chromatography–mass spectrometry

A powerful separation and analysis method employs a combination of gas chromatography (GC) and mass spectrometry (MS), aptly named GC–MS [101, 102]. First, the GC aims to separate the drug mixture. If successful, each component including active ingredients and cutting agents may be isolated and analyzed via MS. MS is considered to be a primary characterization technique [103, 104] that can identify molecules based on their mass and fragmentation pattern. This means of analysis therefore has an advantage over the previous spectroscopic techniques, which all rely on detecting the characteristic pattern of molecular vibrations in a sea of signals associated with every molecule in a mixture. GC–MS is well known for being used as a confirmatory testing technique [8, 12, 85]. In many cases, these are experienced analytical laboratories and technicians working in collaboration with drug checking services to help validate point-of-care results from less sensitive techniques like IR or Raman spectroscopy. Portable GC–MS has been evaluated in forensic applications [105, 106], and in drug checking for the detection of trace substances [27], noting the additional complexities such as maintenance and method development required for such method.

#### Basic principles

**Fig. 8:**
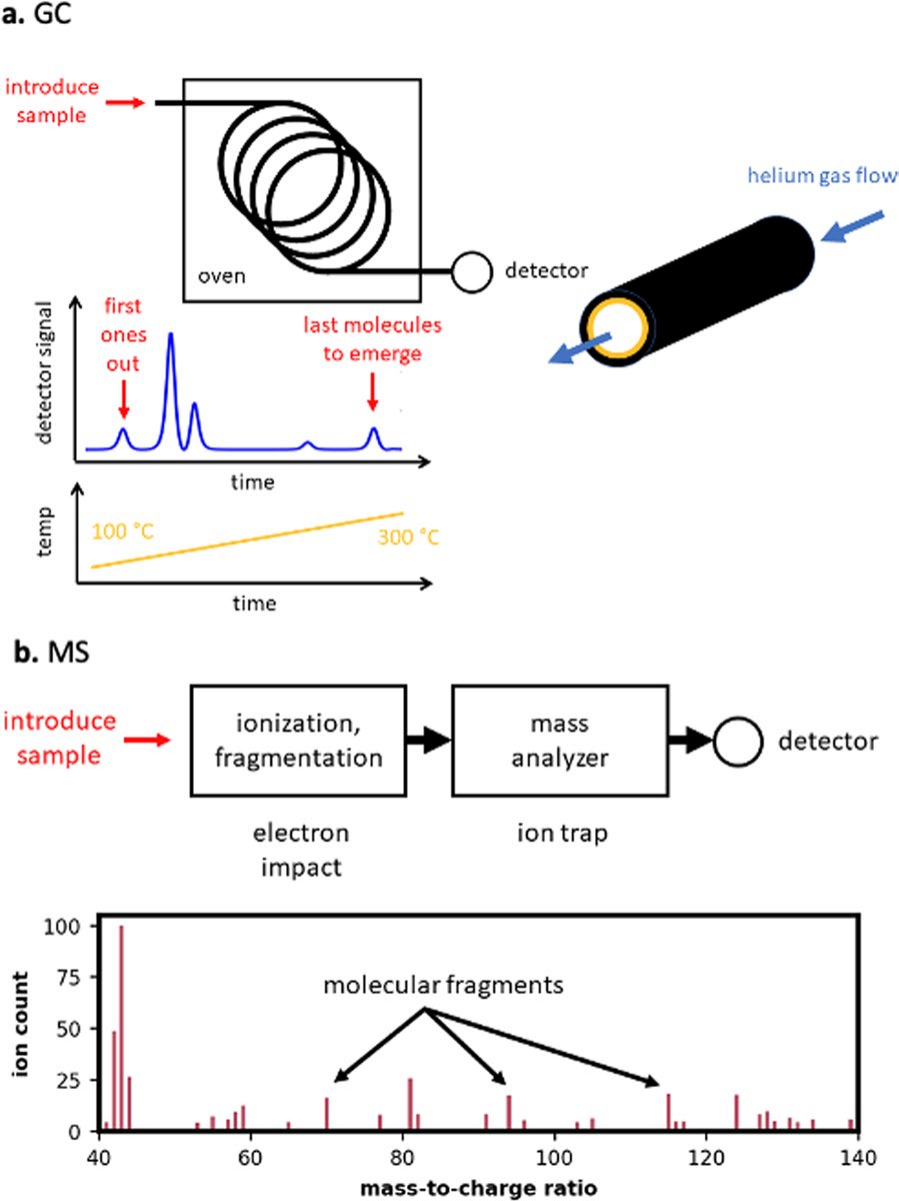
**a** In a gas chromatograph (GC), a small quantity of the sample mixture in injected into the end of a coiled tube inside an oven. Components in the mixture travel through the tube at different speeds, thereby achieving a separation of molecules for easier identification. **b** In a mass spectrometer (MS), molecules are ionized, fragmented, and then detected according to their mass and charge. A GC–MS combines gas chromatography and mass spectrometry technologies into a single instrument, with the GC output flowing directly into the MS for analysis

The general principle of gas chromatography [107] (GC) is illustrated in Fig. 8a. The sample is first dissolved in a volatile solvent such as methanol and then extracted into a fiber probe from either the vapor headspace above the liquid, or by immersing the probe directly into the solution [108]. The fiber is then inserted into a heated injection port, with the temperature set high enough to vaporize the components as they desorb from the fiber and enter the instrument. A carrier gas, typically helium, carries the mixture of drug molecules through a long tube (called a column) that is coiled to be more compact and fit into an oven [109]. The constituent molecules initially travel together, but increasing separation occurs as different molecules experience different degrees of attraction to the inside of the column. All molecules spend some of their time sticking to the inner walls of the column, and some of their time detached from the column. When detached, they are pushed further along the column by the helium carrier gas. Eventually, the molecules that spend the least time attached to the walls emerge at the end of the column first, while those that experience the greatest attraction come out last [110]. A plot of the output count against time (referred to as a chromatogram, Fig. 8a) may be used to identify species based on the characteristic time at which they emerge, termed the retention time. This information is compiled using known standards, and observing their travel through the column. The temperature of the oven that surrounds the column determines the retention time of each species. Finer control of the separation (to increase speed and enhance separation of components with otherwise similar retention times) is achieved by ramping the temperature during the separation, a technique known as temperature programming (Fig. 8a).

In mass spectrometry, a sample mixture is imparted with a charge in a process known as ionization. The molecular structure may be preserved in this process (soft ionization) or the molecule may be broken into fragments [111]. Molecular ions or fragments then enter a mass analyzer and detector that can resolve the mass-to-charge ratio characteristic of a particular molecule as shown in Fig. 8b. In the case of fragments, the particular fragmentation pattern may be pieced together to identify the molecule from which it originated [112]. In theory, no libraries are necessary for molecular identification; the fragments may be related directly to the chemical structure. The use of databases, however, greatly accelerates this identification. MS is a trace analysis method, and so can detect low concentrations of components in a mixture. Nevertheless, analysis of a trace compound in a complex matrix using MS alone is often challenging particularly where highly sensitive detection is desired in a complex sample matrix [107], for much the same reason as mixture analysis complicates the interpretation of IR and Raman data. As shown in Fig. 8c, the output of the GC column may be sent directly into a mass spectrometer to achieve the combined GC–MS.

#### Practical considerations

Portable GC–MS is useful as a point-of-care drug checking instrument as it offers trace-level identification. However, it has a relatively high instrumental and operational complexity when compared to the other instruments discussed so far.

**Table:** 

Why can someone detect only one compound on IR and then see 10 or more peaks on GC–MS?	
This is largely related to the detection method as well as the improved resolution from having chromatographic separation. High temperatures can also result in degradation products which could explain unexpected peaks on a chromatogram– it might be unclear whether it is truly a breakdown product or from the drug mixture. The means that one can inject a pure compound into the GC–MS, and several defined peaks are identified on the chromatogram. In addition, with such sensitive techniques, cross-contamination is a potential risk even in trace amounts.

**Table:** 

Why do some compounds not show up on GC–MS even if they are present in high enough concentrations?	
Some common cutting agents such as sugars have high boiling points and therefore are not volatilized to a large extent, and do not enter the column in appreciable quantities [107]. Some drug molecules are not suitable for identification with GC–MS given that they do not ionize, have limited solubility in the selected solvent, or ultimately do not have a unique retention time or mass spectrum for subsequent identification.	

Too high of a concentration injected into the instrument or improper temperature programming can also be detrimental resulting in significant tailing, overlapped peaks, and saturation effects in the mass analyzer and detector [113]. For drug samples, this requires a balance between preparing a high enough concentration to detect potent, trace drugs such as carfentanil, but not too high to have significant such unfavorable effects from the high amount of cutting agents such as caffeine. Careful method development, such as adjusting the concentration, internal standards, gas flow rate, and temperature programming, can result in reliable and sensitive drug checking.

**Table:** 

Is GC–MS quantitative?	
Yes. Quantification with GC–MS depends on the relationship between the peak area of a specific analyte and the concentration. Within a certain range of concentrations this relationship is typically linear and therefore simple to model. However, parameters outlined above, such as gas flow rate, temperature programming, tailing, injection errors, changes in the mass spectrometer and column bleed will all affect this signal [107].	

Common to many analytical instruments, quantification with GC–MS often uses external standards to create a calibration curve to model this relationship while accounting for some of the instrumental influences on signal through replicate measurements [114]. This model can be used to quantify future unknown samples. However, with GC–MS often the column, detector, and other instrumental factors will also change over time and using an absolute signal intensity can be misleading without re-calibration [115]. Spiking a solution with an internal standard with a fixed and known concentration helps further account for such run-to-run variability [115]. It is important that the internal standard behaves as similar as possible to the drug molecule you are trying to quantify. Other sophisticated methods exist, such as isotope dilutions [116] and the standard addition methods (previously mentioned as methods to aid in SERS quantification) [107]. In either case, accurate and precise results depend on significant method development and validation for each target molecule, as well as accurate weighing and dilution of the drug sample.

**Table:** 

Do we need both the GC and the MS components for drug checking?	
Technically, no. However, GC–MS offers a two-fold advantage over either method employed individually (GC or MS). First, molecules may be unambiguously identified on the basis of both their retention time and fragmentation pattern: any molecules with similar retention times will likely have different mass spectra. Second, the MS analysis is no longer of a mixture, but of a single component from that mixture, greatly simplifying the identification and quantification effort and improving its reliability.	

## Illustrations from an active drug checking service

### Methods

#### Sample selection

Since 2018, the Vancouver Island Drug Checking Project has operated a free and confidential service in Victoria, Canada. Prior to 2021, every sample presented was analyzed using immunoassay test strips, attenuated total reflection (ATR) IR-absorption spectroscopy, Raman spectroscopy from powder samples, surface-enhanced Raman scattering (SERS) in a solution of colloidal gold nanoparticles, and GC–MS—all with portable instruments. In operating the service, we aim to analyze the data in order to report the components present in the drug mixture and, where possible, offer some information on the strength of the drugs. At the same time, we aim to evaluate these portable technologies in the context of community drug checking. We purposively selected four samples of drug mixtures that best illustrate the various degrees of challenge in the analysis with multiple instruments. The samples include a methamphetamine sample containing dimethylsulfone, a cocaine sample containing phenacetin and levamisole, and two different opioid samples. Although samples such as these four repeatably show up at the drug checking service, our selection was not based on their prevalence. Rather, the data were chosen as it most clearly demonstrates many of the principles we have introduced within the background sections and aims to connect concepts such as overlapping IR signals, fluorescence interference in Raman spectroscopy, tailing on GC–MS, etc., to the real application of drug checking as a public health response.

#### Test strips

We use fentanyl (20 ng/mL cut-off) and benzodiazepine (300 ng/mL cut-off) test strips (BTNX, Markham, Canada). In the case of fentanyl test strips, a small amount of substance (1–2 mg) is dissolved in approximately 2 mL of water and manually agitated until dissolved. Fentanyl test strips are run on all samples presented to the drug checking service. When substances known to be MDMA or methamphetamine are presented, a larger volume of water (about 10 mL) is used due to false positives for fentanyl at concentrations greater than 1 mg/mL [54]. Benzodiazepine test strips are used for testing expected opioids, fake Xanax tablets, unknown substances, in cases when a benzodiazepine is suspected, or upon request. Here, approximately 2 mg of sample is placed in 1–2 mL of warm water and agitated until dissolved before using the test strip according to manufacturer instructions.

#### IR spectroscopy

We employ a portable FTIR spectrometer (Agilent 4500a, Agilent Technologies, Santa Clara, California) equipped with a dTGS detector and a single-bounce diamond ATR accessory. Approximately 1–2 mg of sample is used to cover the ATR crystal. FTIR data are initially collected by co-adding 32 scans over the 650–4000 cm$^{-1}$ range at a spectral resolution of 4 cm$^{-1}$. An open-source drug library, SWGDRUG, is used in the analysis of acquired spectra.

#### Raman and SERS

Raman spectra were acquired using a handheld surface offset Raman spectrometer (SORS) (Resolve, Agilent Technologies, Santa Clara, California), equipped with an 830-nm laser. Scattered light with Stokes shifts in the range 200–2000 cm$^{-1}$ is measured directly from a powder sample. About 2–3 mg of sample (more if available) is placed on a disposable aluminum tray prior to measurement. Liquid solutions may also be measured through a glass vial. We use the same instrument for surface-enhanced Raman, but with the sample region fitted with an accessory that enables a glass vial to be inserted and held in the focus of the laser. A small amount of substance (approximately 1 mg or less) is dissolved in 1.5 mL of a 50-nm nanoparticle gold solution (BBI solutions, Crumlin, UK) and shaken for 30 s. If the sample does not appear visibly dissolved, the vial may be briefly heated in a warm water bath. 10 µL of a concentrated (1.0 M) MgSO$_4$ solution is then added and shaken for an additional 10 s before acquisition.

#### GC–MS

The data we show below used the Torion T-9 portable GC–MS (Perkin Elmer, Utah), equipped with a low thermal mass capillary gas chromatograph, in-trap electron impact ionization source, miniaturized toroidal ion trap mass analyzer, and an electron multiplier detector with a mass range of 41–500 Daltons. Approximately 1 mg of a drug sample was dissolved in 150 µL of methanol and centrifuged prior to sampling. A coiled microextraction (CME) syringe was immersed into the sample solution for approximately 10 s and allowed to dry for 1–2 min prior to injection into the GC–MS. Resulting chromatograms were analyzed by selecting or integrating the mass spectra of eluting peaks and comparing to MS libraries from SWGDRUG and NIST databases. In cases where in-house analytical references exist, targeted analysis of compounds may be achieved using a reconstructed ion chromatograph (RIC) with one or more ion fragments of interest. Any resolved peaks are then compared to the retention time of analytical standards and their corresponding mass spectra.

### Results

#### Sample 1: methamphetamine & dimethylsulfone

**Fig. 9:**
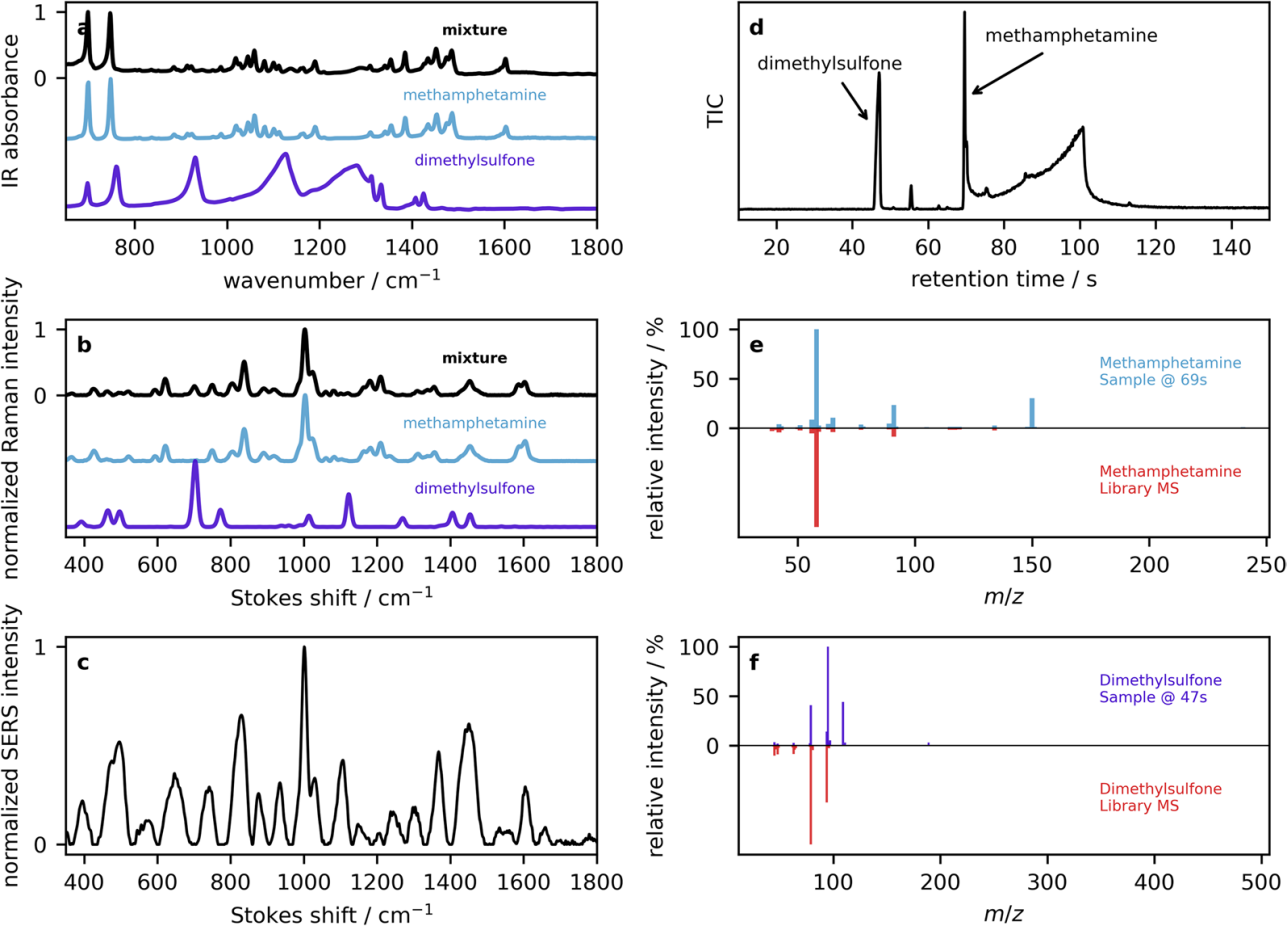
Example 1: **a** ATR–IR reflection absorbance spectra of a sample containing methamphetamine (black), overlaid with library spectra of pure methamphetamine and dimethylsulfone. **b** Raman spectra and the corresponding library entries. **c** SERS spectrum of the drug mixture. **d** The GC total ion count chromatogram is presented, along with mass spectra of **e** methamphetamine and **f** dimethylsulfone in the top panels, and library MS entries below in red

The first example is that of a drug mixture that contains only a single active as the major component and a smaller amount of a single cutting agent, with a negative fentanyl test strip result. Figure 9a shows the ATR–IR spectrum of the drug mixture (black trace) along with the library entry of methamphetamine (blue) that was identified based on its score using a correlation coefficient. Visually, one can see that the spectrum of the mixture closely resembles the spectrum of pure methamphetamine, implying that the drug is primarily methamphetamine. A weighted subtraction of the methamphetamine spectrum enables the residual to be compared against library entries again, this time producing a hit for dimethylsulfone (MSM, purple trace). Although there is little visual evidence for MSM in the spectrum of the mixture, this accounts for some of the broad features near 1300 cm$^{-1}$ and the sharper feature near 925 cm$^{-1}$. A similar analysis was carried out by Raman spectroscopy from the crystalline sample. As mentioned previously, although IR and Raman spectroscopy probe the same characteristic molecular vibrations, the relative intensity of the peaks in the spectra is often different thereby providing a complementary fingerprint. For example, the sharp MSM feature with a Stokes shift just above 700 cm$^{-1}$ (purple trace in Fig. 9b) is more apparent in the Raman spectrum, even at the same concentration. The SERS spectrum is shown in Fig. 9c. As a result of the variability of the SERS spectra, and their specificity for the detailed nature of the substrate, aggregating agent, and solution conditions, SERS libraries need to be tailored for the specific device and measurement protocol. However, it is common to interpret SERS spectra qualitatively in comparison with Raman library entries. One challenge associated with solution-based SERS is the large background associated with additives such as the citrate stabilizer that accounts for many of the modes seen in Fig. 9c. However, one can see the emergence of the 1000 cm$^{-1}$ methamphetamine band. The GC–MS data is first presented in the form of a chromatogram using the MS total ion count (TIC) in Fig. 9d. Several peaks are visible with retention times up to 120 s. Integrating the MS in small windows (approximately $\pm 2$ s) around each peak enables the average MS to be displayed an analyzed in comparison to MS library entries. The MS serves as definitive compound identifications, although they are not strictly required in cases where the characteristic retention times for species are already known. The MS for the 70 s peak is shown in Fig. 9e (blue trace, top), and has a good match to the methamphetamine library spectrum (red trace, bottom). The MS obtained by integrating the 47 s peak is shown in Fig. 9f (purple, top) and agrees with the MSM library entry (red, bottom). Returning to the TIC chromatogram in Fig. 9d, one notices that additional peaks are present. For example, the feature at 55 s has been tentatively identified as benzyl chloride, a suspected breakdown product of methamphetamine or a leftover precursor from the methamphetamine synthesis. As noted, molecular degradation (and the resulting mass fragments observed) due to thermal breakdown is a common occurrence with GC–MS and generally possess additional challenges in the interpretation, but may be helpful as a further indication of a component of interest. One also notices the prominent signal that ends with a sharp edge at 100 s; such tailing is commonly observed for methamphetamine and, however, adds the challenge of potentially obscuring peaks at longer retention times.

#### Sample 2: cocaine & phenacetin

**Fig. 10:**
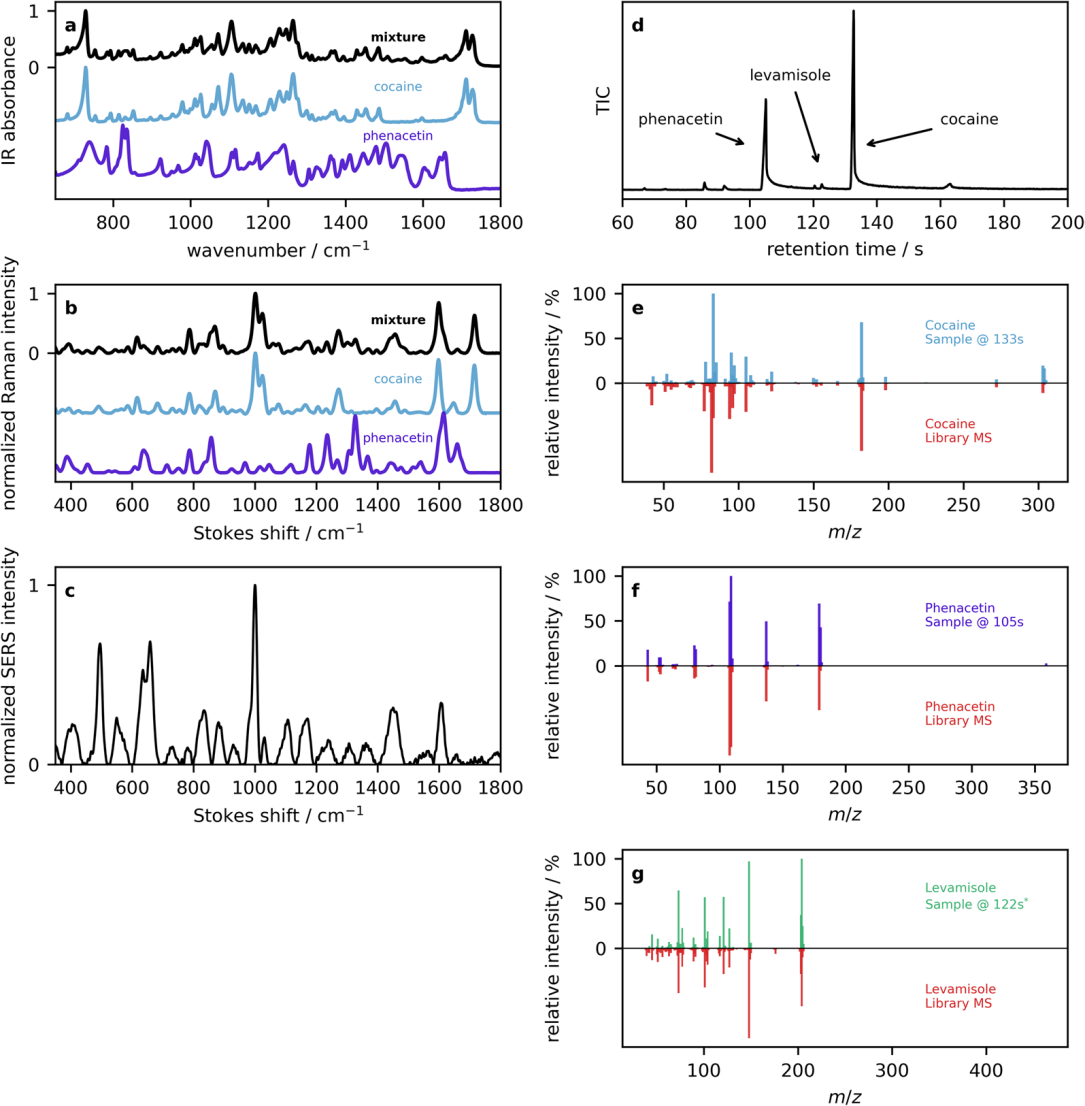
Example 2: **a** ATR–IR reflection absorbance spectra of a sample containing cocaine (black), overlaid with library spectra of pure cocaine and phenacetin. **b** Powder Raman spectra and the corresponding library entries. **c** SERS spectrum of the drug mixture. **d** The GC total ion count chromatogram is presented, along with mass spectra of **e** cocaine, **f** phenacetine, and **g** levamisole in the top panels, and library MS entries below in red

The second example is that of a drug mixture that contains primarily cocaine and phenacetin, again with a negative fentanyl strip test. The ATR–IR spectrum is shown in Fig. 10a (black trace), along with the top library hit of cocaine (blue trace), and the library spectrum of phenacetin (purple) that was obtained by searching the residual following cocaine subtraction. Evidence of phenacetin in several places in the drug mixture, including the two peaks near 820 cm$^{-1}$, and two peaks around 1650 cm$^{-1}$. Although the fingerprint region of phenacetin is more complicated than that of MSM in the previous example as a result of the larger molecular size, this also provides additional details for comparison in the library searching. In the Raman spectra (Fig. 10b), phenacetin peaks are again apparent, this time in the sharp feature at 1340 cm$^{-1}$, and the broad 1620 cm$^{-1}$ shoulder on the sharp 1600 cm$^{-1}$ cocaine peak. The SERS spectrum shown in Fig. 10c displays enhancements at 650 cm$^{-1}$ and 1000 cm$^{-1}$ attributed to cocaine [117]. The largest peaks in the TIC chromatogram (Fig. 10d) at 133 s and 105 s have mass spectra corresponding to cocaine (Fig. 10e) and phenacetin (Fig. 10f). Investigation of minor peaks in the TIC chromatogram reveal that the feature eluding at 122 s has a mass spectrum that matches levamisole (Fig. 10g), a medication used to treat parasitic worm infections, but can have detrimental health effects resulting in serious infection with chronic use and high doses [118]. Levamisole historically has been used as a popular cutting agent in cocaine [118]. It is also noted that cocaine has many synthetic byproducts that appear in the TIC chromatogram.

#### Sample 3: simple opioid mixture

**Fig. 11:**
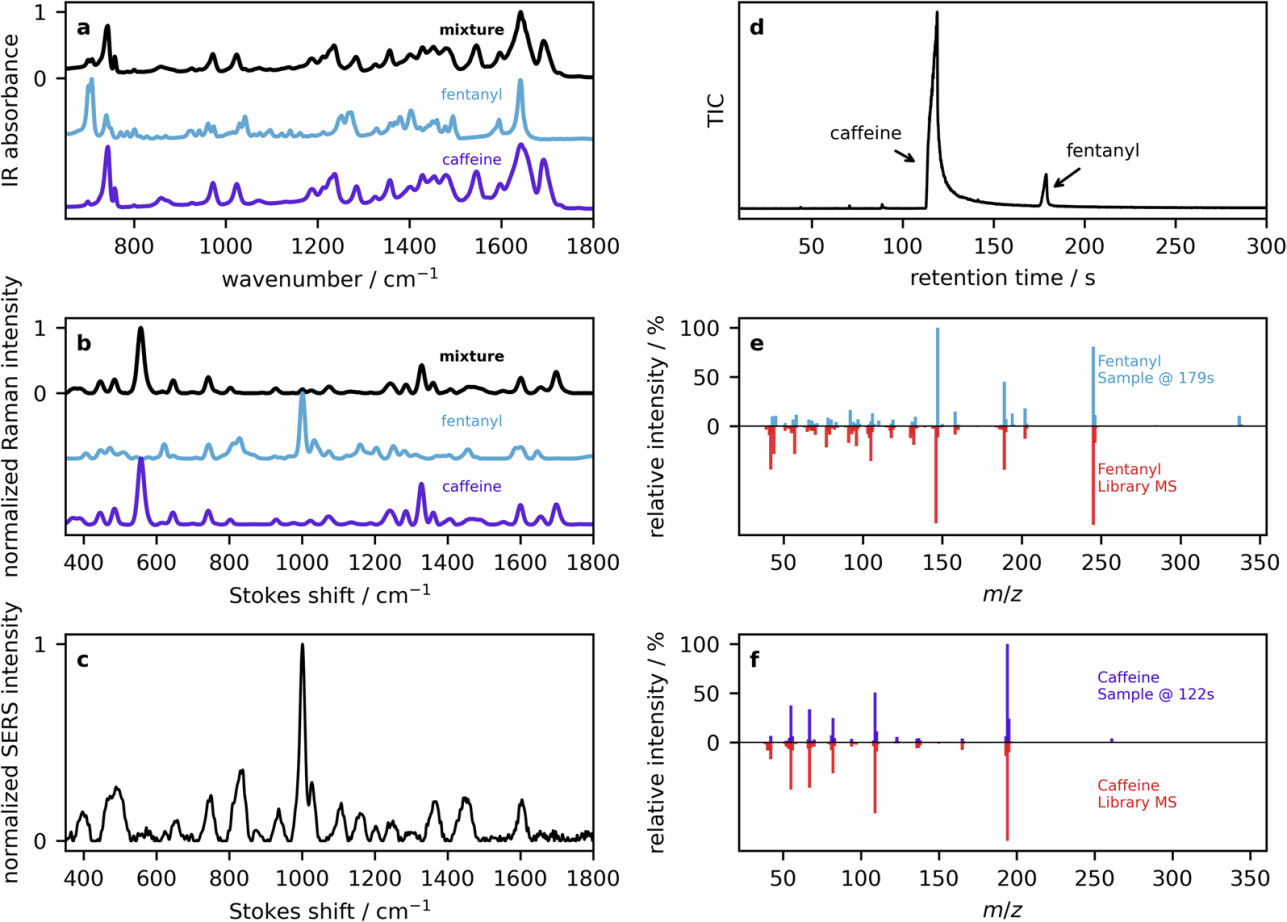
Example 3: **a** ATR–IR reflection absorbance spectra of an opioid sample (black), overlaid with library spectra of pure fentanyl and caffeine. **b** Powder Raman spectra and the corresponding library entries. **c** SERS spectrum of the drug mixture. **d** The GC total ion count chromatogram is presented, along with mass spectra of **e** fentanyl and **f** caffeine in the top panels, and library MS entries below in red

The third example is an opioid sample with positive fentanyl test strip and negative benzodiazepine test strip results. The IR absorption (Fig. 11a) and Raman (Fig. 11b) spectra both show strong correlations with caffeine, the major component in many down samples. Both spectra have minimal visual evidence of fentanyl. However, fentanyl features (most prominently 705 cm$^{-1}$ in the IR and 1000 cm$^{-1}$ in the Raman) can be picked up by library searching. When those interpreting the spectral data have significant domain-knowledge, such well-known peaks are located visually to support library searching algorithms. As opioid samples are commonly presented for analysis in a community drug checking service, we have also developed quantification models based on PLS regression that can be used to estimate the fentanyl concentration [11, 15]. As a safeguard, a novelty detection algorithm can be applied to ensure that the sample has sufficient similarity to the training data used in the development of the models. When our models [11] are applied to the IR spectrum (black trace in Fig. 11a), we obtain a fentanyl concentration of 12% by weight. Applying the Raman model [15] to the data (black trace in Fig. 11b) results in a concentration of 7%. The SERS spectrum shown in Fig. 11c has the same enhancement of the 1000 cm$^{-1}$ mode as seen in the previous two examples, but this time attributed to fentanyl. It is well known that many molecules produce strong Raman signals at 1000 cm$^{-1}$ so this peak can be diagnostic even though it lacks specificity. In other words, SERS may be useful for determining low concentration actives, but one cannot distinguish fentanyl or cocaine from methamphetamine based on this peak alone. The GC–MS total ion chromatogram (Fig. 11d) displays two prominent peaks with constituent mass spectra that are well matched to fentanyl HCl at 179 s (Fig. 11e) and caffeine eluding at 122 s (Fig. 11f). This helps confirm the specific fentanyl analogue that may be ambiguous based on the test strip results and IR or Raman spectra.

In this example, spectroscopic instruments IR and Raman were able to rule-in the correct components (e.g., fentanyl or analogue and caffeine). However, communicating such result based on those instruments alone remains complex due to the inability to rule-out certain trace compounds. While the more sensitive techniques of SERS and GC–MS do not contribute a significant amount of additional compounds to the interpretation, the ruling out of certain compounds such as carfentanil and benzodiazepines is an invaluable piece of information.

#### Sample 4: Complex opioid mixture

**Fig. 12:**
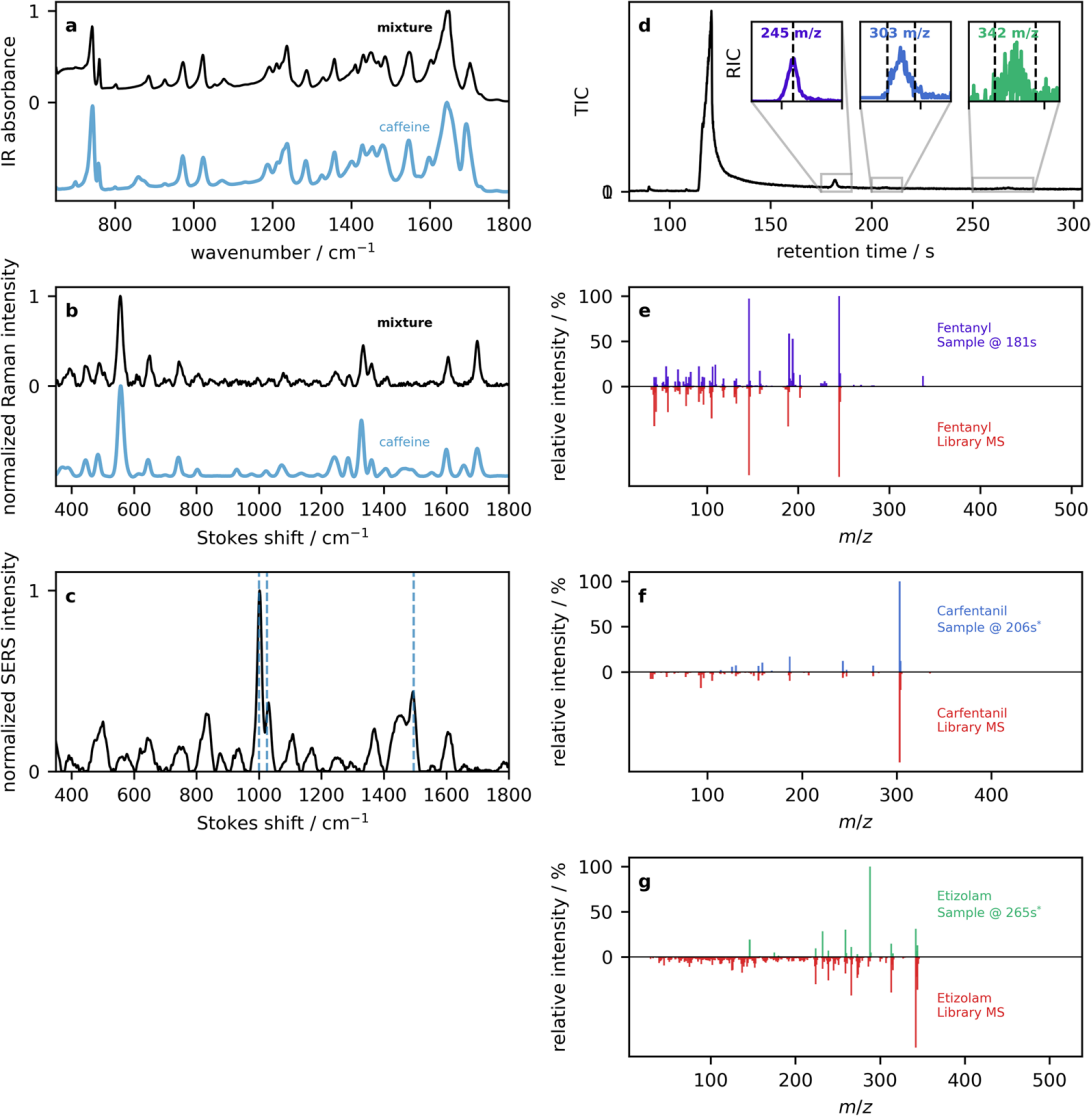
Example 4:**a** ATR–IR reflection absorbance spectra of an opioid sample (black), overlaid with the library spectrum of caffeine. **b** Powder Raman spectra and the corresponding library caffeine spectrum. **c** SERS spectrum of the drug mixture. **d** The GC total ion count chromatogram is presented, along with mass spectra of **e** fentanyl, **f** carfentanil, and **g** etizolam in the top panels, and library MS entries below in red

The last example is that of a challenging opioid sample with a positive fentanyl strip test, and negative benzodiazepine test. Looking at the IR (Fig. 12a) and Raman (Fig. 12b) spectra, the visual appearance and quantitative library match to caffeine is high in both cases, but residual searching does not produce any convincing additional results.

Although IR and Raman have been shown to be capable of detecting fentanyl or other compounds at the 1–2% level, in this case, there are complicating factors that interfere with the analysis. In the IR spectra, a telltale sign of fentanyl at low concentrations (below reliable quantification) is a small peak that appears at 705 cm$^{-1}$. The spectra in Fig. 12a have their caffeine peaks shifted in that region, attributed to the moisture content of the sample [119]. In the case of the Raman, this sample contained a high level of background fluorescence that degraded the signal-to-noise. However, the SERS spectrum in Fig. 12c shows fentanyl at 1000 cm$^{-1}$ and another peak at 1500 cm$^{-1}$ that is characteristic of etizolam and not present in the previous example where only caffeine and fentanyl were identified [16, 120]. Confirmation of low concentration actives comes from the GC–MS data. The total ion chromatogram in Fig. 12d displays a large caffeine peak eluting at 122 s, a small but prominent peak at 181 s, and some minor baseline fluctuations that are barely noticeable and would therefore not be picked up in an untargeted analysis. Targeted analysis can be performed by looking for a specific mass fragment, and therefore species, of interest. This is called a reconstructed ion chromatogram (RIC). The inset to Fig. 12d shows RIC traces for the 245 m/z fentanyl, 303 m/z carfentanil, and 342 m/z etizolam ions. The corresponding MS data obtained by integrating these peaks as shown in Fig. 12e–g. In this case, less sensitive spectroscopic methods were not sufficient to identify compounds in low concentrations that are essential to people who use opioids. Some points regarding the test strips are warranted. First, the lack of specificity to a large number of fentanyl analogues means that a positive result can indicate a wide range of potency based on fentanyl analogues alone. Second, a false negative screen for benzodiazepine-related compounds is common when etizolam is present in a drug mixture [16, 55]. As mentioned previously, this lack of positive strip test in the presence of etizolam (i.e., false negative) can mostly be attributed to the structural diversity within benzodiazepines and benzodiazepine-related substances targeted with the test strips; some molecules will have poorer affinity for the immunoassay than others and therefore higher cut off limits [55]. In this case of a complex opioid mixture, IR and Raman alone are insufficient to account for all components and more sensitive technologies like SERS and GC–MS are needed for a full diagnostic.

## Discussion

Prior qualitative research from the authors has confirmed that drug checking implementation will be limited unless designed to specifically address the barriers of criminalization and stigmatization [6]. The benefits of drug checking must outweigh the risks of accessing the services [6]. As a harm reduction intervention, we argue that drug checking holds potential for impact beyond an individual level, on the unregulated drug market as well as at the community and policy level [2, 121]. However, public health and harm reduction organizations lack clear guidance to inform decisions on which instruments to purchase [122]. They currently do not have sufficient opportunities to assess what the operation of such instruments would actually look like in practice, with numerous potential benefits and limitations to consider. Indeed, researchers studying the implementation of drug checking in a Boston harm reduction service coined the situation to be the “Bronze Age” of drug checking [29]. We concur and contribute new discussions addressing current questions based on practice-based evidence from the Vancouver Island Drug Checking Project in Canada. Our four case studies illustrate three significant issues; the present challenges and opportunities for drug checking instrumentation, the crucial need to communicate uncertainty and limitations, and the beneficial role for confirmatory checking when utilizing more affordable portable instruments.

### Challenges and opportunities for multi-instrument drug checking

In the context of increasingly complex substances and unprecedented levels of illicit drug overdose, there are significant advantages to a multi-instrument approach to drug checking [31, 33, 123]. Using more than one method for drug identification has advantages in terms of increased confidence when a particular component is detected by more than one technique, and increased awareness when something is detected by at least but perhaps only one technique. The first three examples have illustrated scenarios where any of the methods employed are able to detect all of the major components. This is the case when the drugs are relatively uncut or the components are present in high concentrations as in the case of methamphetamine and dimethylsulfone. The cocaine and phenacetin example is similar, except that trace levamisole was not detectable by infrared or Raman spectroscopy. From this perspective, a rapid optical characterization using IR or Raman would be sufficient to confirm that the class of drug suspected is correct. This is generally true for opioid samples as well, as their major components caffeine and/or sugars are easy to detect. The challenge arises when actives are present at levels below the limit of quantification, or even below the limit of detection, of IR or Raman spectroscopy. In the most challenging situation of low-concentration actives, all portable instruments are limited in their detection. However, even in those cases, a few subtle clues from multiple sources may be enough to inform harm reduction messaging and practices. For opioid samples, such challenges are often encountered for fentanyl alone, and almost always encountered in the case of highly potent fentanyl analogues such as carfentanil or adulterants such as benzodiazepines. An interesting circumvention of this limitation is to consider situations where certain technologies may be useful in a majority of cases. For example, opioids are rarely seen at music festivals [74] where the ideal technology is one that can provide rapid testing and results for drug classification when there is high demand. In such cases, portable IR or handheld Raman is an ideal technology, while GC–MS would be too time-consuming. On the other hand, a community drug checking initiative typically receives a significant amount of opioid samples where the classification is often confirmed by a fentanyl strip test, and the more detailed analysis of interest to the service user is both challenging (as in Example 4) and essential due to low concentration but highly potent actives circulating in the drug supply.

### Communicating uncertainty and service limitations

Regardless of what instrument is employed, the recognition and understanding of the sources of uncertainty and limitations is at the forefront of providing a safe and reliable service model. Pragmatic knowledge translation is vital to establishing a trustworthy service [122]. For many service providers the reality is that current out-of-the-box technology is not meeting their needs [29], particularly in the level of nuance and expertise required for the interpretation of the data. Clearly, additional research and development is needed in collaboration with existing and prospective service users and providers. Even before current drug checking technologies existed, service providers monitored the supply, witnessed the effects of the local supply, and understood and advocated for the needs of their community [5, 29, 35, 124, 125]. Integrating instrument knowledge allows the drug checking community to better consider questions such as: What is possible? What isn’t possible and why? What are the fundamental challenges, and can we overcome them?

### Role of confirmatory testing

We have focused our discussion around portable instruments being explored for community drug checking. However, confirmatory laboratory-based testing continues to be an integral part of an effort to provide accurate, sensitive, and quantitative results [126]. Our service utilizes paper-spray mass spectrometry to fulfill this objective [127, 128]. Mass spectrometry data also furthers our research goals to enhance the capabilities of portable instruments as it enables us to benchmark their performance and pursue further method development [16, 27]. This includes machine learning models to help automate and improve spectral analysis, target lists for GC–MS library screening, and developing strategies for communicating known limitations of individual techniques.

## Conclusions

Currently, no single instrument achieves all of the objectives of drug checking. Multi-instrument approaches to drug checking may be required to effectively respond to the growing expectations placed on drug checking services. That is to provide comprehensive, accurate trace-level detection and quantification in a timely and accessible manner. Case studies providing practice-based evidence illustrate some limitations when seeking to directly compare and select instruments for drug checking as a harm reduction response. Comparisons of different technologies demand implementation evidence as well as consideration of the different contexts and demands. An understanding of the principles behind relevant drug checking technologies will help to link the raw data they produce and the results that are communicated to people who use drugs. We hope that the technical foundations we have provided in this perspective will enable the harm reduction community to continue to guide innovations in drug checking.

## Data Availability

All data generated or analyzed during this study are included in this published article.

## Abbreviations

ATR: Attenuated total reflection
ATR–IR: Attenuated total reflection infrared
FT: Fourier transform
FTIR: Fourier transform infrared
GC: Gas chromatography
GC–MS: Gas chromatography mass spectrometry
IR: Infrared
LC–MS: Liquid chromatography mass spectrometry
LOD: Limit of detection
LSD: Lysergic acid diethylamide
MDMA: 3,4-Methylenedioxymethamphetamine
MS: Mass spectrometry
MSM: Dimethylsulfone
NMR: Nuclear magnetic resonance
PLS: Partial least squares
PSMS: Paper spray mass spectrometry
RIC: Reconstructed ion chromatogram
SERS: Surface enhanced Raman spectroscopy
SORS: Surface offset Raman spectroscopy/spectrometer
TIC: Total ion count
